# Chlorotoxin Fused to IgG-Fc Inhibits Glioblastoma Cell Motility via Receptor-Mediated Endocytosis

**DOI:** 10.1155/2012/975763

**Published:** 2012-12-05

**Authors:** Tomonari Kasai, Keisuke Nakamura, Arun Vaidyanath, Ling Chen, Sreeja Sekhar, Samah El-Ghlban, Masashi Okada, Akifumi Mizutani, Takayuki Kudoh, Hiroshi Murakami, Masaharu Seno

**Affiliations:** ^1^Department of Medical and Bioengineering Science, Graduate School of Natural Science and Technology, Okayama University, Okayama 7008530, Japan; ^2^Cell and Cancer Biology Branch, National Cancer Institute, National Institutes of Health, Bethesda, MD 20892, USA; ^3^Department of Pathology, Tianjin Central Hospital of Gynecology Obstetrics, Tianjin 300100, China

## Abstract

Chlorotoxin is a 36-amino acid peptide derived from *Leiurus quinquestriatus* (scorpion) venom, which has been shown to inhibit low-conductance chloride channels in colonic epithelial cells. Chlorotoxin also binds to matrix metalloproteinase-2 and other proteins on glioma cell surfaces. Glioma cells are considered to require the activation of matrix metalloproteinase-2 during invasion and migration. In this study, for targeting glioma, we designed two types of recombinant chlorotoxin fused to human IgG-Fcs with/without a hinge region. Chlorotoxin fused to IgG-Fcs was designed as a dimer of 60 kDa with a hinge region and a monomer of 30 kDa without a hinge region. The monomeric and dimeric forms of chlorotoxin inhibited cell proliferation at 300 nM and induced internalization in human glioma A172 cells. The monomer had a greater inhibitory effect than the dimer; therefore, monomeric chlorotoxin fused to IgG-Fc was multivalently displayed on the surface of bionanocapsules to develop a drug delivery system that targeted matrix metalloproteinase-2. The target-dependent internalization of bionanocapsules in A172 cells was observed when chlorotoxin was displayed on the bionanocapsules. This study indicates that chlorotoxin fused to IgG-Fcs could be useful for the active targeting of glioblastoma cells.

## 1. Introduction

Glioblastoma is one of the most malignant and consistently fatal brain cancers in adults. Treatment of glioma remains a challenge largely because of its rapid growth rate and the highly invasive nature of this disease, despite incremental advances in surgical and radiation therapies [1]. Glioma cells are considered to require the activation of matrix metalloproteinase (MMP)-2, which degrades the extracellular matrix (ECM) during invasion and migration [2, 3]. In the central nervous system, membrane type MMP-1 (MT1-MMP) has a more important role than MMP-2 during ECM remodeling, migration, infiltration, and invasion of gliomas [4]. MT1-MMP on cell surfaces is replenished by autodegradation or clathrin-dependent internalization, and its concentration is stabilized by the tissue inhibitor of MMP (TIMP)-2 [5, 6]. Malignant human gliomas express membrane-anchored MMPs and their endogenous TIMPs [7–10]. Many MMP inhibitors have been developed for human clinical trials, but effective candidates have not been obtained [10, 11]. 

Chlorotoxin (CTX) is a 36-amino acid peptide with four disulfide bridges and is derived from *Leiurus quinquestriatus* (scorpion) venom. CTX has been shown to inhibit low-conductance chloride channels in colonic epithelial cells [12]. Several experiments have used CTX to target brain tumors, exploiting its binding affinity to the glioma-specific chloride ion channel complex, MMP-2, and other proteins [13, 14]. Recently, a conjugate of CTX and fluorescent dye was demonstrated to target brain tumors by visualizing cancer foci *in vivo* [15, 16].

Bionanocapsules (BNCs) are artificial hollow nanoparticles composed of the recombinant envelope L protein of hepatitis B virus, which has a specific affinity for human hepatocytes [17, 18]. To confer BNCs a high affinity for the IgG-Fc domain, the pre-S1 region of L protein was replaced with the ZZ motif in protein A derived from *Staphylococcus aureus* [19, 20]. BNCs displaying anti-human EGFR monoclonal antibodies were delivered successfully to glioma cells in a mouse model of brain tumors [19]. EGFR is expressed not only in tumors but also in normal epithelia; therefore, it may not always be feasible to target brain tumors with EGFR. Thus, we designed a CTX peptide fused to the human IgG-Fc domain (CTX-Fc) in this study to establish a more efficient and specific targeting vehicle for glioblastoma cells.

## 2. Materials and Methods

### 2.1. Cell Culture

A human cell line derived from glioblastoma, A172 (RCB2530), was provided by RIKEN BRC through the National BioResource Project of MEXT, Japan. Glioma cells were grown and subcultured in RPMI medium (Sigma-Aldrich, St Louis, MO, USA) supplemented with 10% fetal bovine serum (FBS, PAA Laboratories, Pasching, Austria) in the presence of 100 IU/mL penicillin and 100 *μ*g/mL streptomycin (Nacalai Tesque, Kyoto, Japan). The cells were maintained at 37°C in a humidified incubator with 95% air and 5% CO_2_. 

### 2.2. Construction of Expression Plasmids

The expression plasmids for CTX fused to human IgG-Fcs (CTX-Fcs) were constructed as follows. An oligonucleotide coding for the CTX peptide was synthesized by Operon Biotechnologies (Tokyo, Japan) and cloned into pET28b (Novagen, Darmstadt, Germany). The DNA fragment coding human IgG-Fcs was excised from the plasmid pBO593 (coding with a hinge domain) and pBO807 (coding without a hinge domain, [20, 21]) using the restriction endonucleases, AgeI and NotI, and then ligated to the 3′-end of the CTX coding sequence downstream of a T7 promoter to code a dimeric form of CTX-Fc (D-CTX-Fc) and a monomeric form of CTX-Fc (M-CTX-Fc), respectively. 

### 2.3. Expression and Purification of M/D-CTX-Fcs

*Escherichia coli* BL21 (DE3) pLysS (Novagen) was transformed with expression vectors for M/D-CTX-Fcs. Transformants were grown in 1 L of LB medium containing 50 *μ*g/mL kanamycin and 10 *μ*g/mL chloramphenicol at 37°C. Protein expression was induced by 0.4 mM isopropyl 1-thio-*β*-D-galactopyranoside. After expression induction, the transformants were cultured at 25°C for 16 h, and the bacteria were harvested. Cell pellets were thawed and homogenized in 20 mL of lysis buffer containing 10 mM Tris-HCl (pH 8.0), 10 mM EDTA, 0.2 M NaCl, and 10% sucrose. The inclusion bodies were collected by centrifugation at 12,000 ×g for 20 min. The inclusion bodies were washed three times with 0.5% Triton X-100. The insoluble fraction was resolved in 4 mL of 6 M guanidinium HCl containing 0.1 M Tris-HCl (pH 8.5). The solution was degassed by aspiration while purging the air with nitrogen gas and supplemented with 50 *μ*L of 2-mercaptoethanol. After 1 h incubation at 37°C in a shaking water bath, the mixture was dispersed into a 20-fold volume of refolding buffer containing 10 mM Tris-HCl (pH 8.5), 0.1 M NaCl, and 0.5 mM oxidized glutathione. Refolding was conducted by incubation at 4°C for 18 h. The pH was then adjusted to 7.0 using acetic acid. Insoluble materials were removed by centrifugation at 12,000 ×g for 20 min. The solution containing refolded protein was applied to a cobalt resin column (TALON superflow metal affinity resin, Clontech, Mountain View, CA, USA), after equilibrating with equilibration buffer containing 50 mM phosphate buffer (pH 7.0) and 300 mM NaCl. The column was then washed with equilibration buffer containing 20 mM imidazole and 0.1% Triton X-100. M/D-CTX-Fcs were eluted with elution buffer containing 50 mM phosphate buffer (pH 7.0), 300 mM imidazole, and 300 mM NaCl. The eluted solution was dialyzed three times against phosphate-buffered saline (Dulbecco's formula, hereafter PBS) for 2 h each time. The purity of M/D-CTX-Fcs in the final preparations was assessed by SDS-PAGE, Coomassie Brilliant Blue (CBB) staining, and western blotting.

### 2.4. Preparation of CTX-Fc-BNCs

We mixed 2 nM (10 *μ*g/mL) ZZ-tagged bionanocapsules (ZZ-BNCs) [19] with M-CTX-Fc or human IgG (Sigma-Aldrich) at a ratio of 1 : 20 and incubated them at 4°C for 1 h in PBS. The precipitates were removed by centrifugation at 12,000 ×g for 5 min. 

### 2.5. Enzyme Immunoassay on Cell Surfaces

The enzyme immunoassay (EIA) was designed to evaluate the binding ability of CTX-Fcs to A172 cell surfaces. Each well of a 96-well plate (Greiner Bio-One, Frickenhausen, Germany) was coated with 10% skim milk (Wako Pure Chemical Industries, Osaka, Japan) in PBS at 25°C for 1 h and washed with PBS. Five thousand A172 cells/well were seeded in RPMI medium supplemented with 10% FBS, 100 IU/mL penicillin, and 100 *μ*g/mL streptomycin. After 20 h of culture, the cells were washed three times with PBS and fixed with 4% paraformaldehyde in PBS. The cells were washed three times with PBS, covered with 10% skim milk in PBS at 25°C for 1 h, and then washed three times with PBS. The cells were incubated with M/D-CTX-Fcs in a range of 0–400 nM in PBS at 25°C for 1 h. The cells were then washed with PBS containing 0.1% Tween-20 (PBST), before adding 100 *μ*L of protein A conjugated to horse radish peroxidase (HRP; Sigma-Aldrich), diluted to 1 : 500, and incubated at 25°C for 1 h. The wells were washed three times with PBST, and 100 *μ*L 3,3′,5,5′-tetramethylbenzidine (TMB) Liquid Substrate System (Sigma-Aldrich) was added to test the peroxidase reaction. After 5 min, the reaction was quenched with 50 *μ*L of 0.5 M sulfuric acid, and the absorbance at 450 nm was measured in each well using a microplate reader (SH-9000; Corona Electric, Ibaraki, Japan). Each experiment was performed in triplicate, and the mean values and standard deviations were calculated. 

### 2.6. Wound Healing Assay

Thirty thousand A172 cells were seeded into a 24-well plate in RPMI medium supplemented with 10% FBS, 100 IU/mL penicillin, and 100 *μ*g/mL streptomycin. After 20 h incubation, each confluent monolayer was scratched using a 200 *μ*L plastic pipette tip to create a wounded cell-free area and washed with RPMI medium supplemented with 10% FBS. The cells were incubated at 37°C with M/D-CTX-Fcs in a range of 0–300 nM in RPMI medium supplemented with 10% FBS, 100 IU/mL penicillin, and 100 *μ*g/mL streptomycin and photographed at 0 and 12 h using an inverted microscope CKX41 (Olympus, Tokyo, Japan). The digital images were acquired with a digital camera U-CMDA3 (Olympus) using the imaging program DP2-BSW (Olympus). The distances between the edges of cell-free areas were measured using NIH Image J. The migration length was defined as the change in the distance between 0 and 12 h, which was normalized by the change in the absence of the stimulant.

### 2.7. Cell Migration Assay

The migration of A172 cells was assayed in 24-well plates with 8 *μ*m pore cell culture inserts (BD, Franklin Lakes, NJ, USA). Five hundred microliters of RPMI medium supplemented with 10% FBS were added to each well, and 3 × 10^4^ cells were seeded into each insert. The cells were incubated with M/D-CTX-Fcs in a range of 0–300 nM in RPMI medium supplemented with 1% BSA at 37°C. After 48 h of culture, the insert chambers were removed, and adherent cells on the bottom of each well were counted. The number of migrated cells was normalized by the number of adherent cells in the absence of CTX-Fcs.

### 2.8. Cell Proliferation Assay

The inhibition of cell growth by M/D-CTX-Fcs was evaluated using a 3-(4, 5-dimethylthiazol-2-yl)-2, 5-diphenyltetrazolium bromide (MTT) cleavage assay with A172 cells. The cells were seeded at 5 × 10^3^ cells/well in 96-well plates in RPMI medium supplemented with 10% FBS. After 20 h of culture, M/D-CTX-Fcs in a range of 0–300 nM were added in triplicate, and the cells were further cultured for 48 h. The cells were then exposed to 5 mg/mL MTT in PBS at a final concentration of 1 mg/mL in culture for 5 h. Formazan crystals formed during the incubation period were dissolved overnight at 37°C by adding 10% SDS containing 20 mM HCl. The absorbance was measured at 570 nm. To assess the viability of cells treated with CTX after 48 h incubation with different concentrations of CTX, the wells were washed twice with RPMI medium supplemented with 10% FBS. The cells were further incubated for 24 h in RPMI medium supplemented with 10% FBS. The viable cells were evaluated using the MTT cleavage assay, as described above. 

### 2.9. Confocal Microscopic Observation

For confocal microscopic observation, A172 cells were grown on 18 mm cover slips (Iwaki, Tokyo, Japan) in 12-well plates. The cells were incubated with 30 nM M/D-CTX-Fcs or 30 nM human IgG-Fc domain [20] in PBS containing 1% BSA for 15 min or 1 h at 4°C or 37°C. The cells were washed twice with PBS to evaluate specific binding to cell surfaces. The cells were fixed with 4% paraformaldehyde in PBS, permeabilized with 0.2% Triton X-100, and blocked with blocking solution containing 10% FBS or 1% BSA in PBS. The cells were washed with PBS and incubated with anti-early endosome antigen-1 (EEA-1) antibody (Cell Signaling Technology, Beverly, MA, USA) for 1 h at 25°C followed by Alexa 555-labelled anti-rabbit IgG (Molecular Probes Inc., Eugene, OR, USA) for 30 min at 25°C. The cells were washed with PBS and incubated with FITC-labeled anti-human IgG-Fc antibody (Sigma-Aldrich) for 30 min at 25°C. After further washes, the nuclei were stained with DAPI (Vector Laboratories Inc., Burlingame, CA, USA), and the cells were visualized using a confocal microscope IX81 (Olympus) with Fluoview FV-1000 (Olympus). To observe the binding of BNCs on cells, the cells were incubated with CTX-Fc-BNCs at 37°C for 1 h. The specific binding of CTX-Fcs was further assessed by competition with a CTX (Sigma-Aldrich). In the competitive assay, the cells were incubated primarily with 300 nM CTX in 1% BSA-PBS at 4°C for 20 min, followed by incubation with CTX-Fc-BNCs in the presence of 300 nM CTX at 37°C for 1 h. The cells were washed with PBS and fixed with 4% paraformaldehyde in PBS, permeabilized with 0.2% Triton X-100, and blocked with blocking buffer. The cells were washed with PBS and incubated with anti-human IgG-Fc antibody labeled with fluorescein isothiocyanate (FITC) (Sigma-Aldrich) for 1 h at 25°C. After further washes, the cells were visualized using a confocal microscope LSM 510 Meta (Carl Zeiss, Jena, Germany) equipped with an argon laser having an excitation laser line of 488 nm coupled with a bandpass filter of 505 nm.

### 2.10. Assessment of Internalization of CTX-Fc-BNCs

Cellular uptake of CTX-Fc-BNCs was evaluated. A172 cells in 60-mm dishes were washed three times with ice-cold PBS and incubated with 2 nM (10 *μ*g/mL) of CTX-Fc-BNCs, human IgG-BNCs, or M-CTX-Fc for 1 h at 4°C or 37°C. After incubation, the cells were washed three times with ice-cold PBS to remove unbounded BNCs and were collected by treatment with 0.025% trypsin. After centrifugation at 5000 ×g for 5 min, the supernatant was discarded, and the cell pellet was washed three times with ice-cold PBS. The cells were then lysed in lysis buffer, incubated for 20 min on ice, and sonicated twice. The extracts were clarified by centrifugation at 12,000 ×g for 5 min at 4°C. Twenty microliters of anti-HBsAg microbead suspension were added to the extracts, and this mixture was incubated overnight at 4°C. After centrifugation at 12,000 ×g for 30 s at 4°C, the beads were washed three times in PBS, suspended in Laemmli buffer supplemented with *β*-mercaptoethanol, heated for 5 min at 95°C, and subjected to SDS-PAGE followed by western blotting. 

### 2.11. Western Blotting and Image Analysis

Proteins resolved on SDS-PAGE were transferred to a polyvinylidene difluoride (PVDF) membrane (Millipore, Billerica, MA, USA). The membrane was blocked with 10% skim milk in 10 mM Tris-HCl (pH 7.4), 150 mM NaCl containing 0.1% Tween-20 (TBST). The blots were probed with anti-human IgG mouse monoclonal antibody conjugated with HRP (Life technologies, Carlsbad, CA, USA) diluted to 1 : 500 in TBST containing 10% skim milk. The HRP signal was developed using a Western Lightning Plus-ECL chemiluminescence reagent (PerkinElmer, Waltham, MA, USA), and the intensities of the bands were visualized using a Light-Capture II cooled CCD camera system (ATTO, Tokyo, Japan). The relative intensities of the blots were quantitatively analyzed using NIH Image J.

### 2.12. Statistical Analysis

The results were expressed as means ± standard deviations from at least three independent experiments. The data were analyzed using Student's *t*-test. *P* < 0.05 was considered statistically significant.

## 3. Results

### 3.1. Preparation of M/D-CTX-Fcs

**Figure 1:**
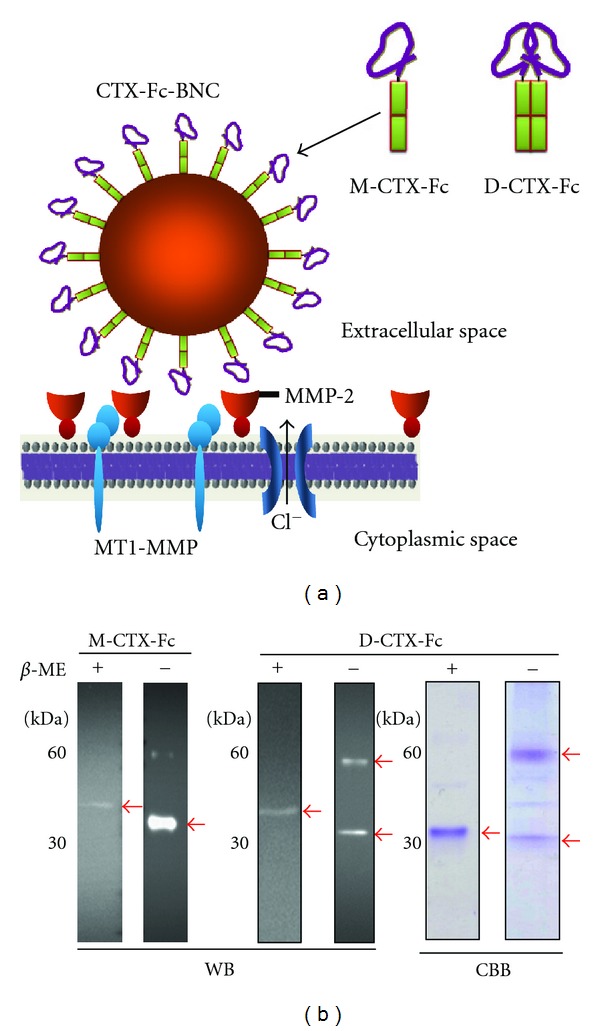
Design and preparation of M/D-CTX-Fcs. (a) Schematic diagrams of monomeric and dimeric CTX-Fcs and the multivalent display of M-CTX-Fc on the surface of ZZ-BNCs. (b) Reduced and nonreduced forms of M/D-CTX-Fcs. M/D-CTX-Fcs were subjected to SDS-PAGE and western blotting. Anti-human-IgG-Fc antibody reacted with the purified M/D-CTX-Fcs without significant degradation. Arrows indicate the purified protein. *β*-ME: beta-mercaptoethanol; WB: western blotting; CBB: Coomassie Brilliant Blue staining.

Schematic representations of M/D-CTX-Fcs and ZZ-BNCs displaying M-CTX-Fcs are shown in Figure 1(a). The His-tagged CTX-Fc fusion protein was designed as a CTX peptide fused to the amino terminus of the human IgG-Fc domain with/without a hinge domain. The CTX-Fcs expressed in *E. coli* were observed as monomers of approximately 30 kDa under the reducing condition, whereas CTX-Fcs with a hinge domain were observed as dimers of approximately 60 kDa under the nonreducing condition, which was confirmed using CBB staining or western blotting (Figure 1(b)). 

### 3.2. Intracellular Localization of M/D-CTX-Fcs in A172 Cells

**Figure 2:**
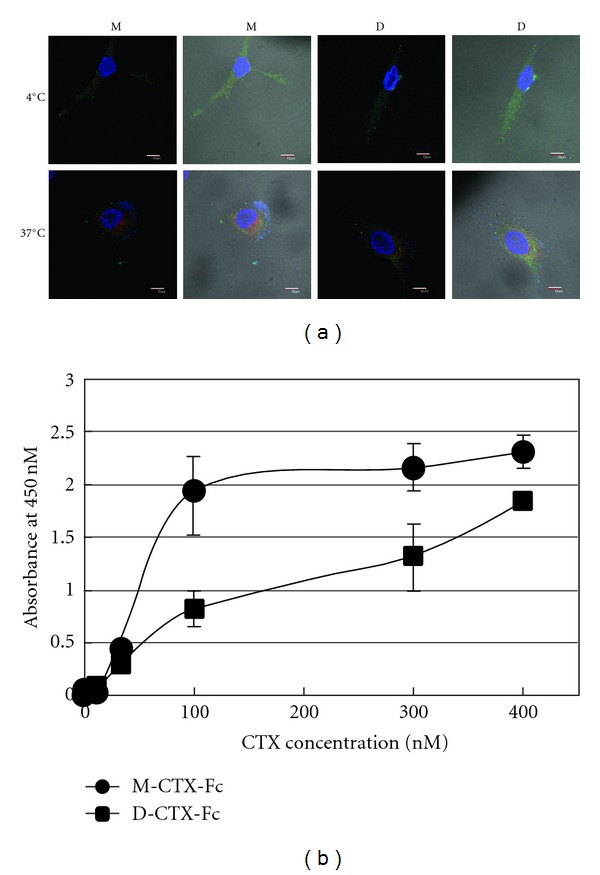
Immunofluorescence image and binding assay for M/D-CTX-Fcs using A172 cells. (a) The M/D-CTX-Fcs attached to cell surfaces at 4°C (upper). Fifteen minutes incubation at 37°C promoted the internalization of M/D-CTX-Fcs into cells (lower). The cells were stained with anti-human IgG antibody labeled with FITC, anti-EEA1 antibody and anti-rabbit IgG antibody labeled with alexa-555, and DAPI. Left: fluorescence image; right; composite image. M: M-CTX-Fc; D: D-CTX-Fc. Bars = 10 *μ*m. (b) The binding ability of M/D-CTX-Fcs was evaluated by EIA. A172 cells were fixed on EIA plates and exposed to M/D-CTX-Fcs. The affinity of M-CTX-Fc for cell surfaces was higher than that of D-CTX-Fc.

Because of the high expression levels of MMP-2 [22], we evaluated the binding capabilities of M/D-CTX-Fcs on the surface of A172 glioblastoma cells. When the cells were incubated with M/D-CTX-Fcs at 4°C, the fluorescence from anti-human IgG labeled with FITC indicated the localization of the fused proteins on the plasma membrane. However, when the cells were incubated at 37°C, the fluorescence indicated that M/D-CTX-Fcs were localized intracellularly in A172 cells (Figure 2(a)). In contrast, the human IgG-Fc domain without a CTX domain produced no fluorescence at 4°C or 37°C indicating the specific binding of the CTX moiety to A172 cell surfaces (see Figure S1 in Supplementary Materials available online at doi:10.1155/2012/975763). We quantitatively evaluated the cell surface binding affinity by assaying sequentially diluted M/D-CTX-Fcs using the A172 cells fixed in EIA plates. The results showed that M-CTX-Fc had a higher affinity than D-CTX-Fc and that 100 nM of M-CTX-Fc saturated the binding (Figure 2(b)).

### 3.3. Effect of M/D-CTX-Fcs on the Migration of A172 Cells

**Figure 3:**
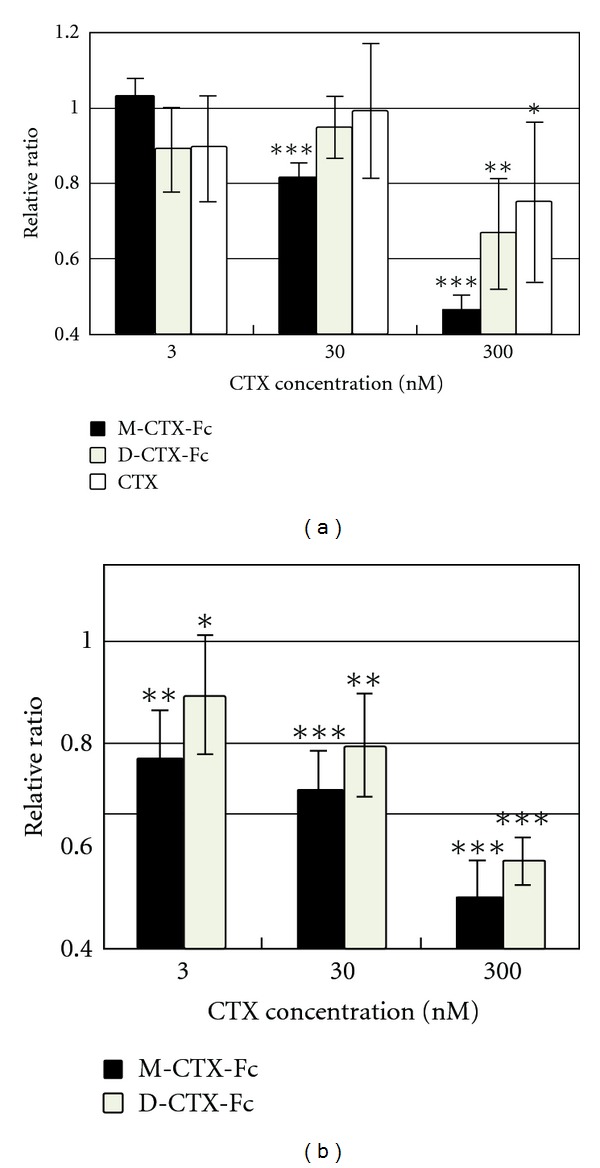
Cell migration and wound healing assays. (a) The effect of M/D-CTX-Fcs on the migration of A172 cells was assessed using a PET track-etched membrane culture insert (pore size, 8.0 *μ*m). The cells were incubated with M/D-CTX-Fcs in the range of 0–300 nM. Translocated cell numbers were normalized against those in the absence of CTX. The results are shown as means ± SD. (b) The inhibition of cell migration by M/D-CTX-Fcs was assayed by wound healing. The effect on migration was evaluated based on the change in the distance. The data (mean ± S.D) presented are from three independent experiments. (*P < 0.1, **P < 0.05, ***P < 0.01).

The effect of M/D-CTX-Fcs on the migration of A172 cells was assessed (Figure 3(a)). Although M-, D-CTX-Fcs, and CTX at a concentration of 300 nM significantly inhibited the migration of the cells, M-CTX-Fc exhibited the inhibition clearly depending on the concentration. In the wound healing assay, the effect of inhibition by both M- and D-CTX-Fcs appeared to be dose dependent in the range of 3–300 nM (Figure 3(b)). The results showed that M-CTX-Fc had a more efficient inhibitory effect than D-CTX-Fc.

**Figure 4:**
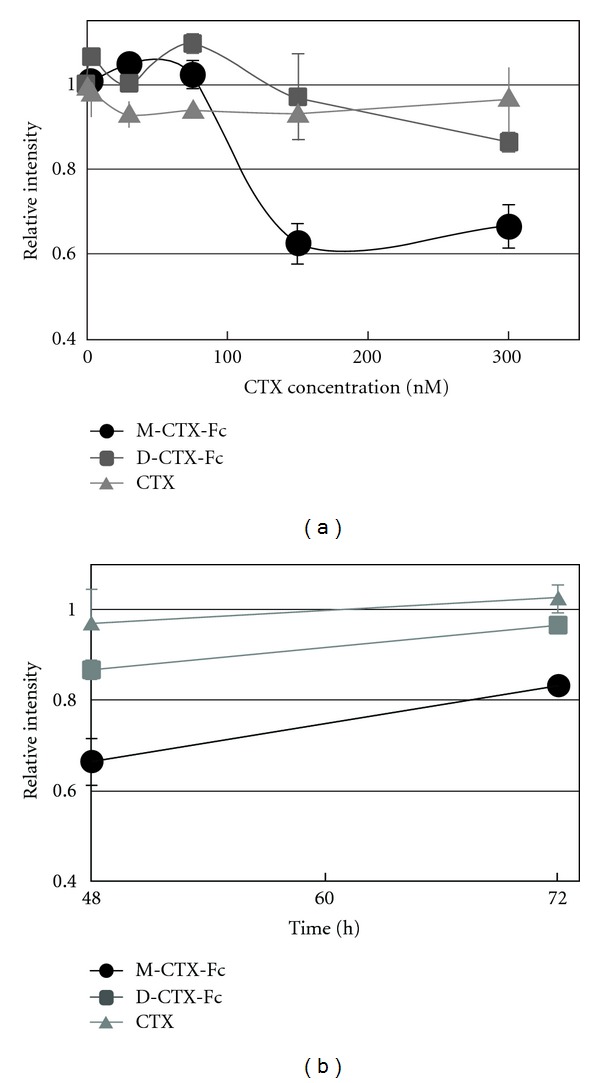
Proliferation inhibition activity. (a) The inhibition of cell growth in the presence of M/D-CTX-Fcs for 48 h. (b) The viable cells at 48 h were kept cultured without M/D-CTX-Fcs up to 72 h. Cell numbers in each well were assessed by MTT assay. The absorbance at 570 nm corresponding to the initial number of the cells was defined as 1.

We then evaluated the effects of M/D-CTX-Fcs on the proliferation and viability of A172 cells. M-CTX-Fc strongly suppressed the cell viability compared with D-CTX-Fc and CTX (Figure 4(a)). IC_50_ was estimated at around 100 nM. After treatment with 300 nM M/D-CTX-Fcs for 48 h, the growth of cells resumed in the next 24 h when the medium was replaced with a medium without M/D-CTX-Fcs or CTX (Figure 4(b)). 

### 3.4. Internalization of CTX-Fc-BNCs

**Figure 5:**
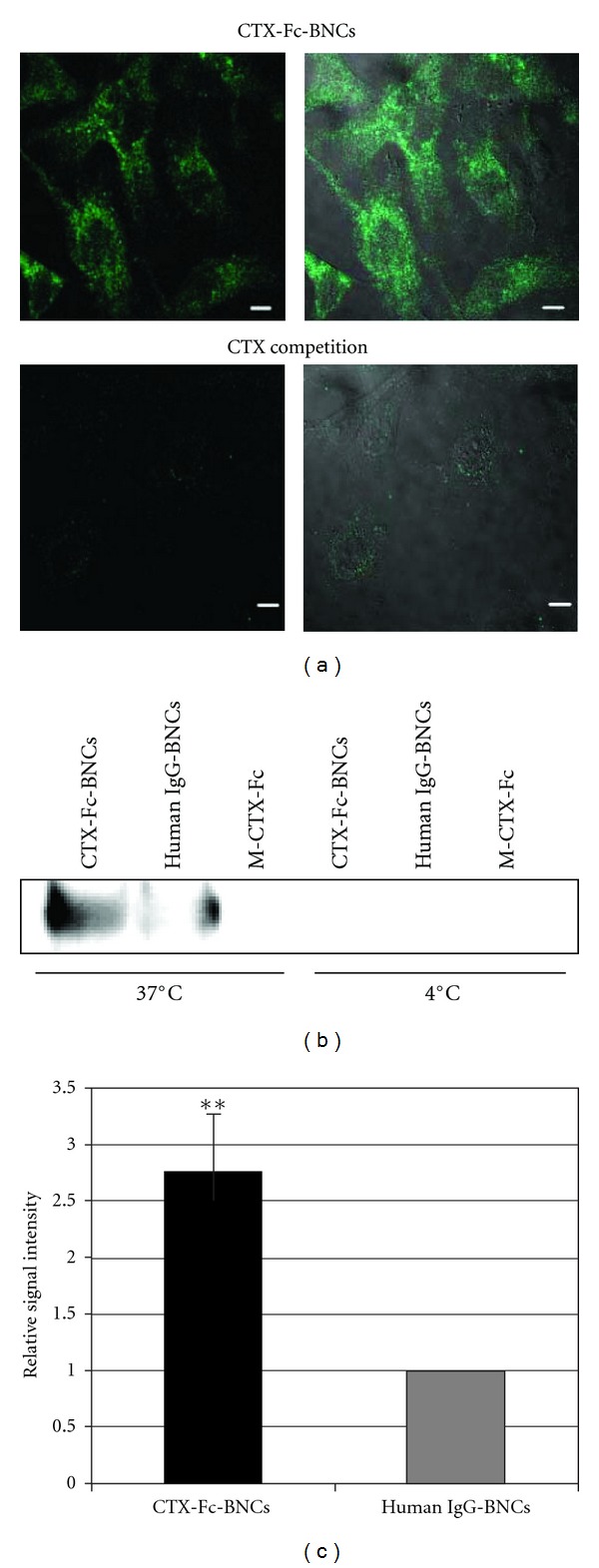
Evaluation of CTX-Fc-BNCs internalized by A172 cells. (a) A172 cells were incubated with CTX-Fc-BNCs at 37°C. In “CTX competition”, the cells were treated primarily with CTX at 4°C for 20 min before incubating with CTX-Fc-BNCs. The cells were stained with anti-human IgG antibody labeled with FITC. Left: fluorescence image; right; composite image. Bars = 10 *μ*m. (b) and (c) A172 cells were treated with CTX-Fc-BNCs, human IgG-BNCs, or M-CTX-Fc for 1 h at 4°C or 37°C. After incubation, the cells were trypsinized. The cytoplasmic fraction was immunoprecipitated with anti-HBsAg antibody conjugated to microbeads. (b) The precipitates were immunoblotted and detected with anti-human-IgG-Fc antibody. (c) The BNC bands in the CTX-Fc-BNCs or human IgG-BNCs treatment at 37°C were analyzed densitometrically using a CS Analyzer 3.0 and plotted in each graph to estimate the amount endocytosed. The data (mean ± S.D) presented are from three independent experiments (**P < 0.05).

The M-CTX-Fc was multivalently displayed on the surface of ZZ-BNCs, thereby exploiting the affinity of the ZZ peptide for the IgG-Fc region [20]. CTX-Fc-BNCs (2 nM, 10 *μ*g/mL) were incubated with A172 cells at 37°C for 1 h, and the specific binding of CTX-Fc-BNCs was observed competing with free CTX (Figure 5(a)). To evaluate the internalization of CTX-Fc-BNCs, the cells were incubated with M-CTX-Fc, human IgG-BNCs, or CTX-Fc-BNCs at 37°C or 4°C. The incubation of cells at 37°C facilitated the intracellular localization of BNCs, indicating that the temperature-dependent internalization was attributable to a membrane-dependent mechanism (Figures 5(b) and 5(c)). 

**Figure 6:**
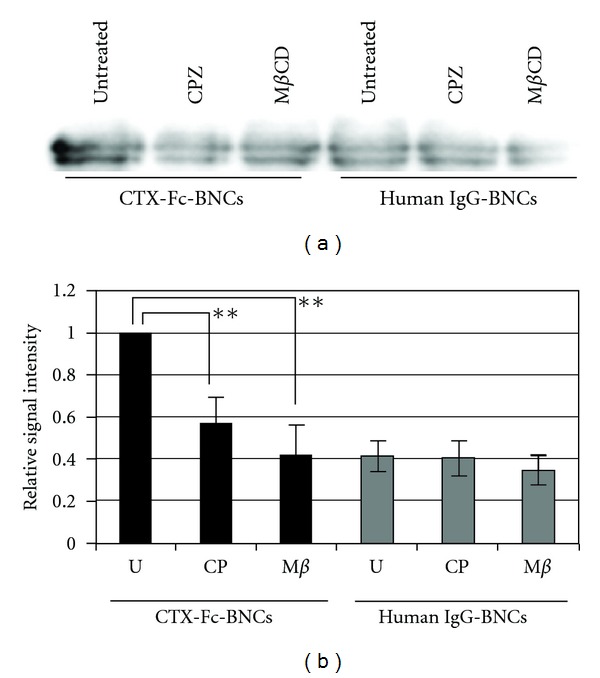
Assessment of the mechanism of CTX-Fc-BNCs internalization. A172 cells were treated with CTX-Fc-BNCs or human IgG-BNCs in the presence of 100 nM CPZ or 5 mM M*β*CD at 37°C for 1 h, followed by trypsinization. The cytoplasmic fraction was immunoprecipitated with anti-HBsAg antibody conjugated to microbeads. “Untreated” indicates that the cells were treated with CTX-Fc-BNCs in the absence of any inhibitors. (a) The precipitates were immunoblotted and detected using anti-human-IgG-Fc antibody. (b) The BNC bands were analyzed densitometrically using a CS Analyzer 3.0 and plotted in each graph to evaluate the amount endocytosed. U: untreated; CP: CPZ; M*β*: M*β*CD. The data (mean ± S.D) presented are from three independent experiments (**P < 0.05).

The mechanism of uptake of CTX-Fc-BNCs was assessed in A172 cells using endocytotic pathway inhibitors (Figure 6). To determine the effective concentration of CPZ, the cells were incubated with 2 nM of CTX-Fc-BNCs and CPZ in the range of 0–100 nM (Vaidyanath et al. 2011 [20], Figure S2). One hundred nanomolar of CPZ effectively inhibited the internalization of CTX-Fc-BNCs in A172 cells. The cells were treated with CPZ, an amphiphilic drug that inhibits the clathrin-mediated pathway, and the internalization of CTX-Fc-BNCs was reduced to the same level as that of human IgG-BNCs. 

## 4. Discussion

Migration of glioma cells is considered to be correlated with MMP-2 expression and activity [2, 3]. Membrane-associated MT1-MMP mediates proteolysis and activates the precursor of MMP-2 (pro-MMP-2), which localizes on the cell surface, and these events occur at the invasive edge of tumor cell nests [6, 23, 24]. Most MMPs have a hemopexin C-terminal domain (C domain), which is linked to the C terminus of the catalytic domain via a flexible proline-rich linker peptide [25–27]. It is considered that MMP-2 contributes to migration, invasion, translocation, and malignancy. In glioma cells, it was reported that CTX inhibits cell invasion by reducing MMP2 activity [13]. In addition, MMP-2 is associated with cell signaling by binding to integrins directly. The proteolytically activated form of the C terminus of MMP-2 can bind integrins on melanoma cells and blood vessels [28]. An angiogenic regulator, angiopoietin 2, induces invasion by stimulating MMP-2 expression and secretion in glioma cells [29]. In cancer, MMPs, such as MMP-2 and MT1-MMP, associate with tumor growth, tissue remodeling, tissue invasion, and metastasis. We designed and purified M/D-CTX-Fcs (Figure 1). M/D-CTX-Fcs were attached to A172 cell surfaces, and they localized intracellularly at 37°C (Figure 2). Furthermore, M/D-CTX-Fcs inhibited cell migration and proliferation in a dose-dependent manner (Figures 3 and 4). Collectively, CTX was shown to inhibit and arrest the cell proliferation machinery but without being toxic to the cells (Figure 4(b)). These findings suggest that M/D-CTX-Fcs may be a potential ligand for the active targeting of glioblastoma cells. 

Several MMPs are considered to regulate signaling pathways in cells [30]. MT1-MMP influences the cellular microenvironment and promotes cell invasion via degradation of ECM, shedding of CD44 and syndecan1, and activation of ERK, Akt, and FAK signaling [31, 32]. MT1-MMP is internalized, and like other membrane-binding molecules, it is regulated by endocytosis because of the functional role of internalization in the cytoplasmic tail [33]. The regulation of the activity and internalization of MT1-MMP are associated with integrin on endothelial cells [34]. Endocytosis and accumulation of MT1-MMP are mediated by the clathrin-dependent endocytic pathway [33]. CTX-Fc-BNCs were localized intracellularly at 37°C (Figure 5), which was inhibited by 100 nM CPZ, a blocker of clathrin-coated pit formation [21], and by 5 mM m*β*CD (Figure 6), a cholesterol-dislodging oligosaccharide that inhibits caveolar formation and perturbs clathrin-coated endocytic vesicles [35, 36]. Because 300 nM CTX significantly reduced the green fluorescence of BNCs by competing with CTX-Fc-BNCs (Figure 5(a)), CTX-Fc-BNCs binding on A172 cell surfaces should be specific to the CTX-binding site such as MMP-2 and MT1-MMP. The internalization of CTX-Fc-BNCs was shown to be temperature dependent (Figure 5(b)). This suggests that cellular uptake of CTX-Fc-BNCs was receptor mediated.

Zhang et al. reported that CTX was displayed on polyethylene glycol (PEG-) coated iron oxide nanoparticles that were detectable in the tumor lesions of mouse and rat glioma models. They demonstrated the active targeting of glioma cells using a combination of CTX and supermagnetic or fluorescent compounds *in vivo* and *in vitro* [37–40]. CTX-displaying nanoparticles were able to pass the blood-brain barrier (BBB) or the blood-tumor barrier (BTB) after intravenous injection and accumulated in brain tumors [38, 41]. Many methods, such as intratumoral injection, intracavity injection, microdialysis, biodegradable polymers, and enhanced convection, have been used for local drug delivery to brain tumors [42]. Given the characteristic features of CTX-Fc-BNCs, the targeted intravenous injection of brain tumors with nanodrugs displaying CTX-Fcs should alleviate painful side effects in patients.

## 5. Conclusions

We designed a fusion protein between CTX and human IgG-Fcs. Depending on the presence of hinge region of Fc domain, the fusion protein exists as a monomer or a dimmer. The monomeric form, M-CTX-Fc, performed as an active targeting ligand to suppress the motility of A172 glioblastoma cells. We then constructed a protein nanocapsule displaying M-CTX-Fc as CTX-Fc-BNCs, which showed specific affinity to the surface of A172 cells and internalized into the cytoplasmic space. This internalization depended on the clathrin-mediated endocytosis pathway. Thus the internalization was enhanced by the multivalent display of the ligand on nanocapsules, which should be a promising drug delivery system for targeting glioblastoma when an appropriate anticancer agent is loaded.

## Supplementary Material

Figure S1: Confocal microscopic observation for M/D-CTX-Fcs. The M/D-CTX-Fcs attached to cell surfaces at 4°C (upper). One-hour incubation at 37°C promoted the internalization of M/D-CTX-Fcs into cells (lower). The cells were stained with anti-human IgG antibody labeled with FITC. Left: fluorescence image; Right; composite image. M: M-CTX-Fc; D: D-CTX-Fc; Fc: human IgG-Fc. Bars = 10 *μ*m.
Figure S2: Effect of CPZ on internalization of CTX-Fc-BNCs. A172 cells were treated with CTX-Fc-BNCs in the presence of CPZ in the range of 0–100 nM at 37°C for 1 h, followed by tripsinization. The cytoplasmic fraction was immunoprecipitated with anti-HBsAg antibody conjugated to micro beads. The precipitates were immunoblotted and detected using anti-human-IgG-Fc antibody. The BNC bands were analyzed densitometrically using a CS Analyzer 3.0 and plotted in each graph to evaluate the amount endocytosed.
