# Supplementary figures and images for: Chlorotoxin Fused to IgG-Fc Inhibits Glioblastoma Cell Motility via Receptor-Mediated Endocytosis
Source: J Drug Deliv. 2012 Dec 5;2012:975763. doi: 10.1155/2012/975763 (PMC3523153; doi:10.1155/2012/975763)

**Supplemental Figures**

**Figure S1**

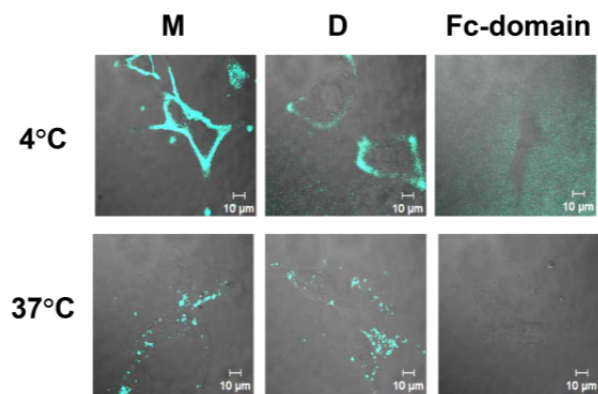

**Figure S2**

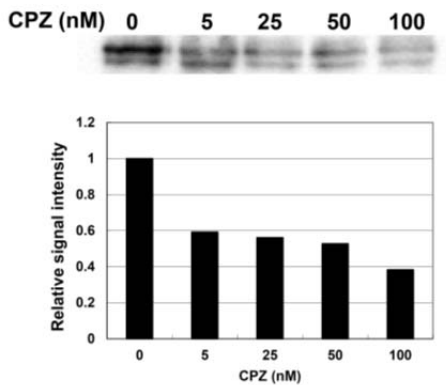

Supplement: Supplementary file 1 — Figure S1: Confocal microscopic observation for M/D-CTX-Fcs. The M/D-CTX-Fcs attached to cell surfaces at 4°C (upper). One-hour incubation at 37°C promoted the internalization of M/D-CTX-Fcs into cells (lower). The cells were stained with anti-human IgG antibody labeled with FITC. Left: fluorescence image; Right; composite image. M: M-CTX-Fc; D: D-CTX-Fc; Fc: human IgG-Fc. Bars = 10 μm. Figure S2: Effect of CPZ on internalization of CTX-Fc-BNCs. A172 cells were treated with CTX-Fc-BNCs in the presence of CPZ in the range of 0–100 nM at 37°C for 1 h, followed by tripsinization. The cytoplasmic fraction was immunoprecipitated with anti-HBsAg antibody conjugated to micro beads. The precipitates were immunoblotted and detected using anti-human-IgG-Fc antibody. The BNC bands were analyzed densitometrically using a CS Analyzer 3.0 and plotted in each graph to evaluate the amount endocytosed. [file 975763.f1.pdf]
